# Linking a role of lncRNAs (long non-coding RNAs) with insulin resistance, accelerated senescence, and inflammation in patients with type 2 diabetes

**DOI:** 10.1186/s40246-018-0173-3

**Published:** 2018-08-23

**Authors:** Chandrakumar Sathishkumar, Paramasivam Prabu, Viswanathan Mohan, Muthuswamy Balasubramanyam

**Affiliations:** Department of Cell and Molecular Biology and Dr. Rema Mohan High-Throughput Screening (HTS) Lab, Madras Diabetes Research Foundation and Dr. Mohan’s Diabetes Specialties Centre, Gopalapuram, Chennai, 600 086 India

**Keywords:** lncRNA, SASP, HDAC3, Type 2 diabetes, Insulin resistance, Inflammation

## Abstract

**Background:**

Studying epigenetics is expected to provide precious information on how environmental factors contribute to type 2 diabetes mellitus (T2DM) at the genomic level. With the progress of the whole-genome resequencing efforts, it is now known that 75–90% of the human genome was transcribed to generate a series of long non-coding RNAs (lncRNAs). While lncRNAs are gaining widespread attention as potential and robust biomarkers in the genesis as well as progression of several disease states, their clinical relevance and regulatory mechanisms are yet to be explored in the field of metabolic disorders including diabetes. Despite the fact that Asian Indians are highly insulin resistant and more prone to develop T2DM and associated vascular complications, there is virtually lack of data on the role of lncRNAs in the clinical diabetes setting. Therefore, we sought to evaluate a panel of lncRNAs and senescence-inflammation signatures in peripheral blood mononuclear cells (PBMCs) from patients with type 2 diabetes (T2DM; *n* = 30) compared to individuals with normal glucose tolerance (NGT; *n* = 32).

**Results:**

Compared to control subjects, expression levels of lncRNAs in PBMCs from type 2 diabetes patients showed significantly (*p* < 0.05) increased levels of HOTAIR, MEG3, LET, MALAT1, MIAT, CDKN2BAS1/ANRIL, XIST, PANDA, GAS5, Linc-p21, ENST00000550337.1, PLUTO, and NBR2. In contrast, lncRNA expression patterns of THRIL and SALRNA1 were significantly (*p* < 0.05) decreased in patients with T2DM compared to control subjects. At the transcriptional level, senescence markers (p53, p21, p16, and β-galactosidase), proinflammatory markers (TNF-α, IL6, MCP1, and IL1-β), and epigenetic signature of histone deacetylase-3 (HDAC3) were significantly (*p* < 0.05) elevated in patients with type 2 diabetes compared to control subjects. Interestingly, mRNA expression of Sirt1 and telomere length were significantly (*p* < 0.05) decreased in patients with type 2 diabetes compared to control subjects. Majority of the altered lncRNAs were positively correlated with poor glycemic control, insulin resistance, transcriptional markers of senescence, inflammation, and HDAC3 and negatively correlated with telomere length. Logistic regression analysis revealed a significant association of altered lncRNA signatures with T2DM, but this association was lost after adjusting for insulin resistance (HOMA-IR) and senescence markers.

**Conclusion:**

Our study provides a clinically relevant evidence for the association of altered lncRNAs with poor glycemic control, insulin resistance, accelerated cellular senescence, and inflammation.

**Electronic supplementary material:**

The online version of this article (10.1186/s40246-018-0173-3) contains supplementary material, which is available to authorized users.

## Introduction

According to the latest edition of International Diabetes Federation Atlas [1], around 425 million people worldwide have diabetes and India alone harbors more than 73 millions of people with diabetes. While more than 90% of the diabetic population is affected majorly by type 2 diabetes mellitus (T2DM), it is a complex multi-factorial disease involving genetic, epigenetic, and environmental components. Several studies imply that accelerated aging, cellular senescence, and proinflammation are closely linked to the etiology of type 2 diabetes and insulin resistance [2, 3]. Although the association between the proinflammation and senescence in the development of insulin resistance and type 2 diabetes is well known, the underlying molecular mechanisms and upstream regulatory networks are only poorly understood. Epigenetics appears to play a major role in the regulation of inflammation and cellular senescence—the dual pathological features commonly associated with type 2 diabetes [4]. Aberrant epigenetic modifications such as DNA methylation, histone modification, and non-coding RNA alterations are well-recognized drivers for the cancer phenotype, but the accumulating evidence also implies their role in the etiology of diabetes and cardiovascular diseases.

Of the total genome that is transcribed, only 2% codes for proteins, whereas the vast majority of it is transcribed as non-coding RNAs which include long non-coding RNAs (lncRNAs), microRNAs, and others [5]. Of late, lncRNAs have gradually come into the spotlight for the increased appreciation of their functional importance both in health and disease [6]. lncRNAs were also found next to protein-coding genes that are under tight transcriptional control, and often, their expression pattern correlates with tissue differentiation, development, and disease [7]. The widespread dysregulation of lncRNA expression in several disease states and the finding that many lncRNAs are enriched for SNPs that associate with human traits/diseases have highlighted their role as master regulators [8, 9]. Challenging the concept that protein-coding genes are the sole contributors to the development of human disease, recent studies emphasize that lncRNAs mediate disease pathogenesis and hence should be studied and targeted for therapeutic benefits [10]. Accumulating literature on genetic, experimental, and epidemiological studies also highlights a growing list of lncRNAs that control glucose homeostasis and contribute to the pathogenesis of diabetes and its complications. Despite the fact that Asian Indians are highly insulin resistant [11] and more prone to develop T2DM and associated vascular complications [12], there is lack of data on the role of lncRNAs in the clinical diabetes setting and this is the rationale behind our study. Therefore, we planned to study the potential interactions among insulin resistance, cellular senescence, and proinflammation with a central focus on lncRNAs so as to better understand the clinical significance of these molecular perturbations in type 2 diabetes.

## Research design and methods

### Recruitment of the study subjects

Study participants with normal glucose tolerance (NGT; *n* = 32) and patients with type 2 diabetes (T2DM; *n* = 30) were recruited from Dr. Mohan’s Diabetes Specialties Centre, Chennai, India, and from the ongoing epidemiological cohorts. The study was approved by the institutional ethics committee of the Madras Diabetes Research Foundation and conducted according to the principles of Declaration of Helsinki. Written informed consent was obtained from all the study participants prior to the start of the study. All the study participants were clinically well characterized into respective groups according to the World Health Organization (WHO) classification criteria. While all the diabetic patients were on oral hypoglycemic agent (OHA) treatment, < 10% were also on insulin, in addition to OHA.

### Anthropometric measurements

Anthropometric measurements including weight, height, and waist circumference were obtained using standardized techniques. Height was noted down with a tape measured to the nearest centimeter. Weight was measured with traditional spring balance that was kept on a firm horizontal surface. Body mass index (BMI) was calculated using the formula: weight (kg)/height (m^2^). Waist circumference was measured using a non-stretchable fiber measuring tape. Blood pressure was recorded from the right arm in a sitting position to the nearest 2 mmHg with a mercury sphygmomanometer (Diamond Deluxe BP apparatus, Pune, India). Two readings were taken 5 min apart, and the mean of the two readings was represented as the blood pressure.

### Biochemical and clinical investigations

Fasting plasma glucose (glucose oxidase–peroxidase method), serum cholesterol (cholesterol oxidase–peroxidase–amidopyrine method), serum triglycerides (glycerol phosphate oxidase–peroxidase–amidopyrine method), and HDL cholesterol (direct method–polyethylene glycol-pretreated enzymes) were measured using Hitachi-912 Autoanalyser (Hitachi, Mannheim, Germany). The intra and inter assay co-efficient of variation for the biochemical assays was < 5%. Low-density lipoprotein (LDL) cholesterol was calculated using the Friedewald formula [13]. Glycated hemoglobin (HbAlc) was estimated by high-pressure liquid chromatography using the variant analyzer (Bio-Rad, Hercules, Calif., USA). Serum insulin was estimated using enzyme-linked immunosorbent assay (Calbiotech, CA). Insulin resistance was calculated using the homeostasis assessment model (HOMA-IR) using the formula: fasting insulin (μIU/mL) × fasting glucose (mmol/L)/22.5.

### Blood collection and isolation of peripheral blood mononuclear cells (PBMCs)

Fasting blood (5–8 mL) was collected into the vacutainer tube and processed immediately for cell isolation within 2 h from the time of collection. Blood was processed for peripheral blood mononuclear cell (PBMC) isolation using Histopaque-1077 (Sigma-Aldrich) according to the standard protocol by overlaying the blood on density gradient solution and centrifugation at 1500–1800 rpm for 30 min. The buffy coat layer containing the PBMCs was aspirated, washed thrice with phosphate-buffered saline (PBS; pH 7.2–7.4), and aliquoted for various experiments.

### RNA extraction and cDNA synthesis

Total RNA was extracted using TRIzol reagent (Invitrogen) according to the manufacturer’s protocol. RNA quantity and quality were assessed by NanoDrop 2000 (Thermo Scientific) instrument. For the first-strand cDNA synthesis reaction, total RNA (1 μg) was adjusted with nuclease-free water and mixed with the cDNA synthesis master mix containing 100 units of RevertAid M-MuLV reverse transcriptase enzyme and 2× buffer, random hexamer primers (1×), 20 units of RNase inhibitor, and 10 mM dNTP solution mix. The resultant samples were incubated at 42 °C for 60 min for the first-strand cDNA synthesis followed by a 5-min incubation at 95 °C for enzyme deactivation. cDNA reaction negative control without reverse transcriptase enzyme (−RT) was also performed.

### lncRNA/mRNA expression by Q-PCR

A panel of lncRNAs was chosen for this study based on their involvement in metabolic disorders as well as their emerging roles in senescence [14, 15]. The relative expression of the lncRNA/mRNA signatures were analyzed by preparing reaction mixer with FastStart Universal SYBR Green Master (Roche) and the corresponding gene-specific primers (Sigma) with diluted cDNA and final volume made up to 20 μL using nuclease-free water. Quantification and analysis were carried out in LightCycler® 96 real-time PCR System (Roche). The target gene expression was normalized to the house-keeping gene 18SrRNA (lncRNA) and β-actin (mRNA), and relative expression was determined using 2^−ΔΔCT^ method. Non-template control (NTC) was also performed for each reaction assay plate.

### DNA isolation and measurement of telomere length

For the measurement of telomere length, DNA was isolated from the whole blood by phenol–chloroform extraction and ethanol precipitation [16]. Relative telomere length was determined by real-time PCR approach as previously described by Cawthon [17] with a minor modification in the PCR temperature conditions. This method measures the factor by which the ratio of telomere repeat copy number to single-gene copy number differs between a sample and that of a reference DNA sample. PCR amplification was achieved using telomere (T) and single copy gene, 36B4 (encodes acidic ribosomal phosphoprotein) primers (S), which serves as a quantitative control. The mean telomere repeat gene sequence (T) to a reference single copy gene (S) was represented as T/S ratio—a reflection of relative telomere length [3].

### Statistical analysis

All data are represented as mean ± standard error mean (SEM) unless otherwise mentioned as standard deviation (SD). Based on our pilot study on the expression levels of lncRNAs and using the SPSS software, the minimum sample size required for the study was calculated as 28 in each group considering the level of significance set at 0.05 and the statistical power at 0.90. Comparison between groups was performed using the independent sample Student *t* test with *p* < 0.05 as the criterion for statistical significance. Pearson correlation analysis was done between variables and the risk factors. Binary logistic regression analysis was performed to show the association between lncRNAs (independent variable) and diabetes (dependent variable). All analyses were done using SPSS Statistics (version 20.0) and GraphPad Prism (version 6).

## Results

### Clinical and biochemical characteristics of the study groups

Clinical and biochemical characteristics of the study subjects are depicted in Table 1. BMI and waist circumference were slightly and significantly higher in patients with type 2 diabetes compared to control subjects. Patients with type 2 diabetes exhibited significantly (*p* < 0.001) increased fasting plasma glucose and HbA1c compared to the control subjects. T2DM patients were also hyperinsulinemic and insulin resistant as characterized by significantly elevated fasting insulin levels and HOMA-IR values, respectively. Blood pressure and lipid parameters did not differ significantly between the groups.

**Table 1:** Clinical and biochemical characterization of the study subjects

Parameter	Normal glucose tolerance [NGT] (*n* = 32)	Type 2 diabetes mellitus [T2DM] (*n* = 30)	*p* value
Age (years)	44 ± 8	46 ± 8	0.218
Gender—male (female)	18 (14)	18 (12)	**–**
Body mass index (kg/m^2^)	25 ± 3.1	27 ± 4	*0.015*
Waist circumference (cm)	85 ± 8	94 ± 9	*< 0.001*
Fasting plasma glucose (mg/dL)	87 ± 9	136 ± 24	*< 0.001*
Glycated hemoglobin—HbA1c (%)	5.6 ± 0.34	8.1 ± 1.9	*< 0.001*
HOMA-IR	1.8 ± 0.8	6.9 ± 3	*< 0.001*
Fasting insulin (μIU/mL)	8.6 ± 3.5	22 ± 7.2	*< 0.001*
Systolic blood pressure (mmHg)	120 ± 25	131 ± 21	0.079
Diastolic blood pressure (mmHg)	79 ± 13	80 ± 8	0.795
Total cholesterol (mg/dL)	174 ± 28	169 ± 37	0.545
Serum triglycerides (mg/dL)	132 ± 71	138 ± 49	0.737
HDL cholesterol (mg/dL)	41 ± 10	39 ± 7	0.352
LDL cholesterol (mg/dL)	107 ± 21	102 ± 34	0.568
VLDL	27 ± 14	28 ± 10	0.732

Data represented as mean ± SD. Italicized value represents statistically significant compared to NGT

### Altered lncRNA signatures in T2DM

**Fig. 1:**
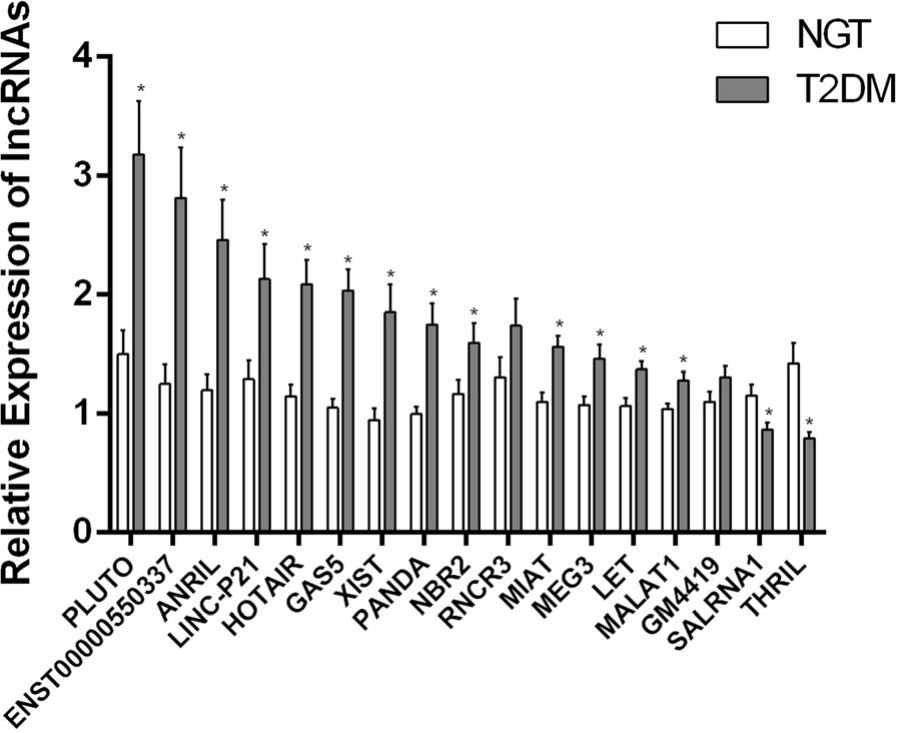
Quantitative real-time PCR analysis of a panel of lncRNA expression levels in PBMCs from the study groups (NGT vs T2DM). Bars represent the mean ± SEM; *p value < 0.05 compared to control subjects

Compared to control subjects, expression profiling of lncRNAs in PBMCs from type 2 diabetes patients showed significantly (*p* < 0.05) increased levels of HOTAIR, MEG3, LET, MALAT1, MIAT, CDKN2BAS1/ANRIL, XIST, PANDA, GAS5, Linc-p21, ENST00000550337.1, PLUTO, and NBR2 (Fig. 1). In contrast, lncRNA expression patterns of THRIL and SALRNA1 were significantly (*p* < 0.05) decreased in patients with T2DM compared to control subjects (Fig. 1).

### Augmentation of HDAC3 and impaired Sirt1 expression in T2DM

**Fig. 2:**
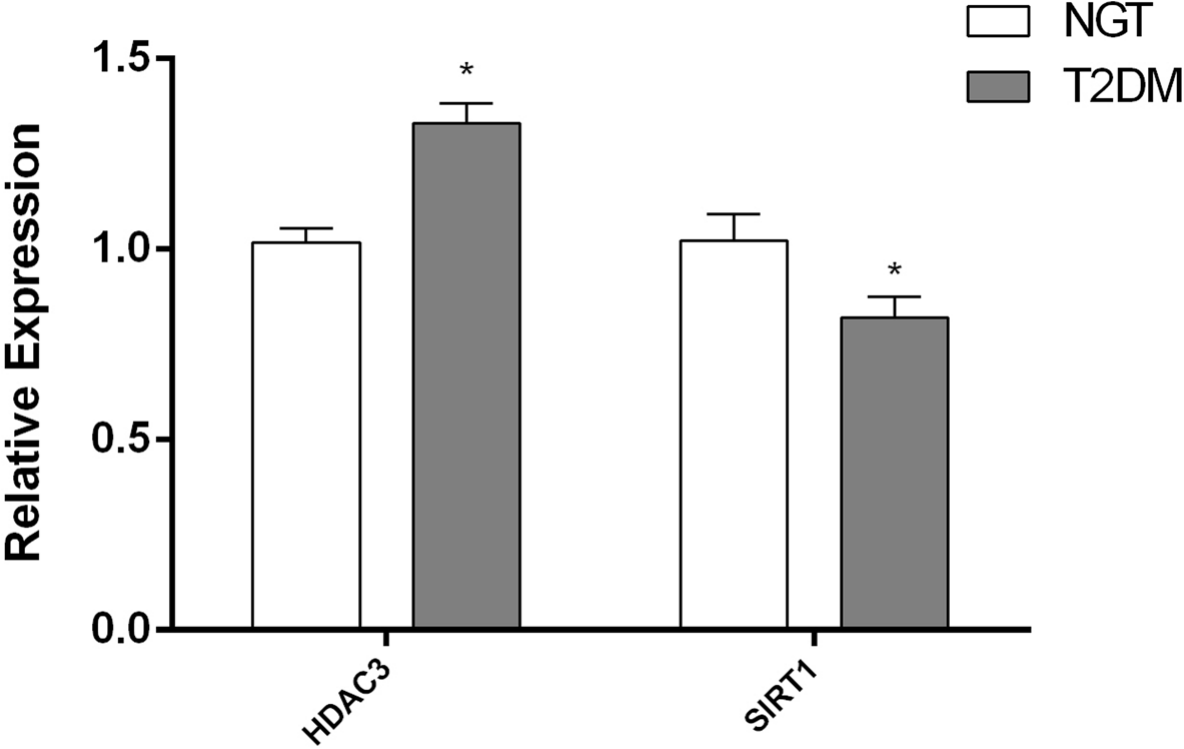
Quantitative real-time PCR analysis of HDAC3 and SIRT1 in PBMCs from the study groups (NGT vs T2DM). Bars represent the mean ± SEM; *p value < 0.05 compared to control subjects

Transcriptional profiling revealed that mRNA expression of HDAC3 was significantly (*p* < 0.05) increased while the Sirt1 level was significantly (*p* < 0.05) decreased in patients with type 2 diabetes compared to control subjects (Fig. 2).

### Altered senescence, inflammation, and telomere length in T2DM

**Fig. 3:**
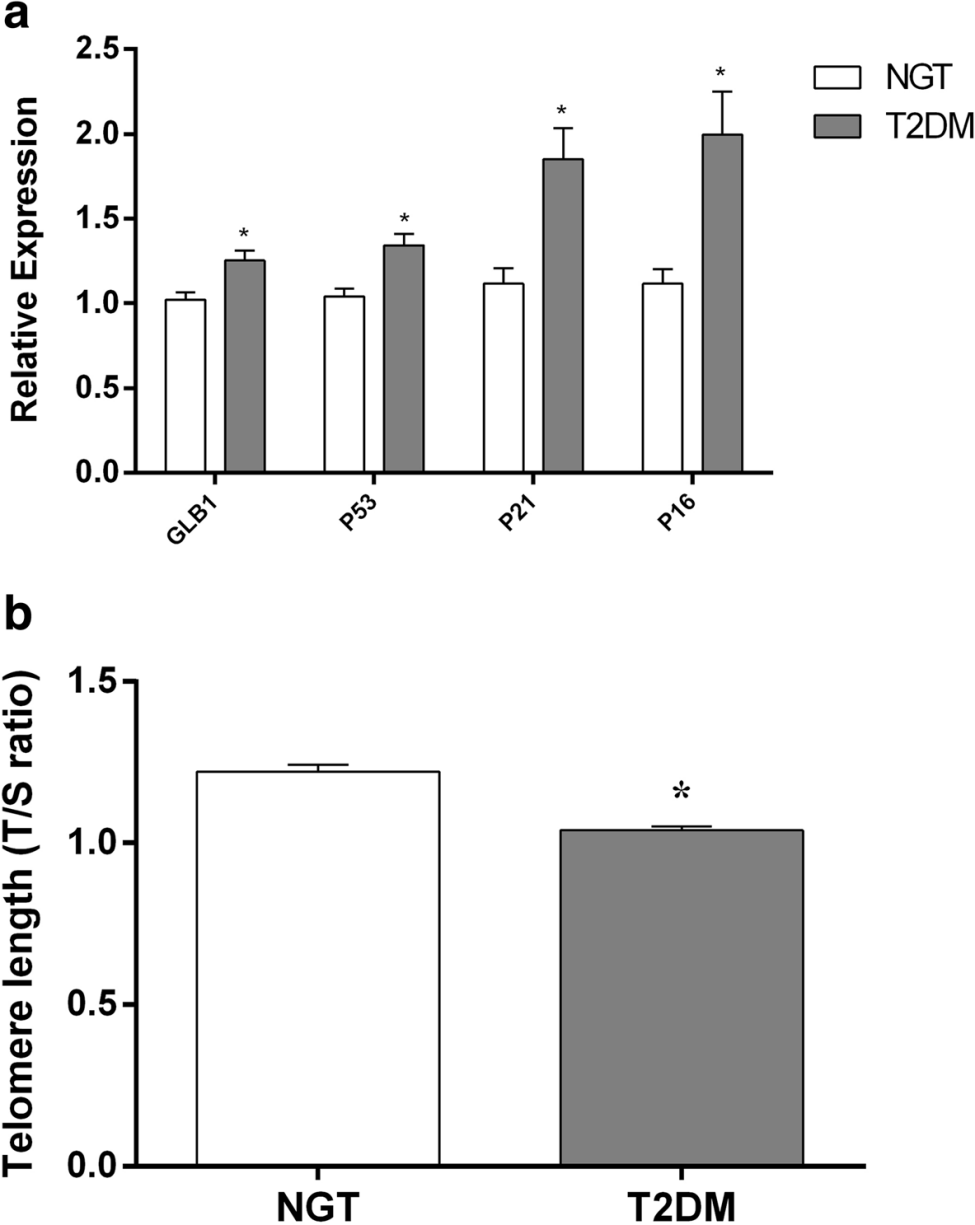
Quantitative real-time PCR analysis of senescence marker gene expression levels, viz., GLB1, P53, P21, and P16 (**a**), and telomere length (**b**) in PBMCs from the study groups (NGT vs T2DM). Bars represent the mean ± SEM; *p value < 0.05 compared to control subjects

**Fig. 4:**
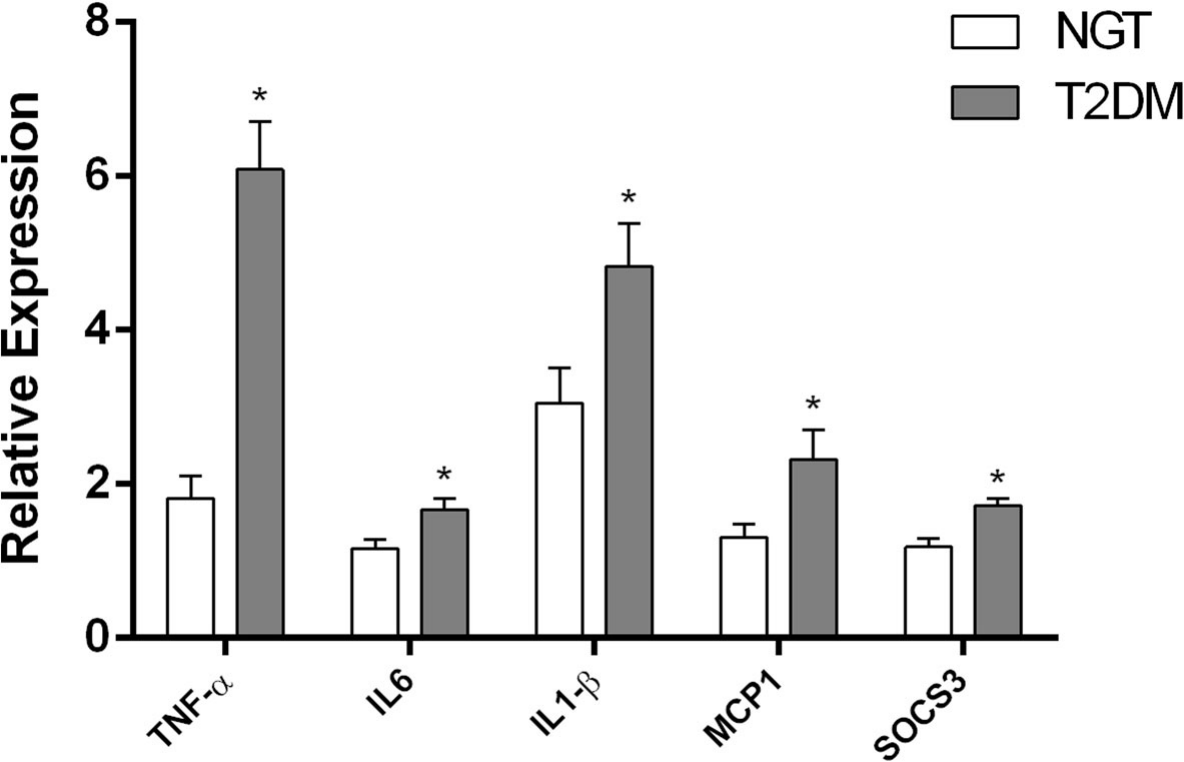
Quantitative real-time PCR analysis of inflammatory signature gene expression levels, viz., TNFα, IL6, MCP1, IL1β, and SOCS3 in PBMCs from the study groups (NGT vs T2DM). Bars represent the mean ± SEM; *p value < 0.05 compared to control subjects

At the transcriptional level, senescence markers, viz., p53, p21, p16, and β-galactosidase 1 (GLB1), were significantly (*p* < 0.05) elevated in patients with type 2 diabetes compared to control subjects (Fig. 3a). As a final read-out of augmented cellular senescence, patients with T2DM were also characterized by significantly (*p* < 0.05) shortened telomeres compared to control subjects (Fig. 3b). Interestingly, mRNA expression levels of proinflammatory gene mediators, viz. TNF-α, IL6, MCP1 and IL1-β, were also significantly upregulated (*p* < 0.05) in PBMCs from patients with type 2 diabetes, implying an acquisition state of senescence-associated secretory phenotype (Fig. 4).

### Correlation analysis

A detailed correlation analysis of lncRNAs with various clinical and biochemical parameters (Additional file 1: Table S1) and molecular parameters (Additional file 2: Table S2) of the study subjects were summarized in the supplement tables. Majority of the altered lncRNAs were positively correlated with poor glycemic control, insulin resistance, transcriptional markers of senescence, inflammation, and HDAC3 and negatively correlated with telomere length. In contrast, expression levels of lncRNAs, viz., SALRNA1 and THRIL, were negatively correlated to glycemic control, insulin resistance, markers of senescence, inflammation, and HDAC3 and positively correlated to telomere length.

### Logistic regression analysis

Logistic regression analysis using type 2 diabetes as dependent variable revealed that altered expression levels of lncRNAs, viz., PLUTO, ENST00000550337.1, CDKN2BAS1, LincRNA-P21, HOTAIR, GAS5, XIST, PANDA, NBR2, MIAT, MEG3, LET, MALAT1, SALRNA1, and THRIL, were associated significantly with T2DM, and this statistical significance was persisted even after adjusting for confounding factors like age and BMI. Interestingly, this statistical association was lost when adjusted for HOMA-IR and senescence markers. This implies that the association between lncRNAs and T2DM could be closely linked to insulin resistance and accelerated senescence with downstream inflammatory signaling (Table 2).

**Table 2:** Binary logistic regression analysis using type 2 diabetes as dependent variable

	Unadjusted		Adjusted for age and BMI		Adjusted for HOMA-IR		Adjusted for senescence markers (GLB, P53, P21, P16, and TL)		Adjusted for inflammatory markers (TNF-α, IL6, MCP1, IL1-β, and SOCS3)	
	*β*	*p*	*β*	*p*	*β*	*p*	*β*	*p*	*β*	*p*
PLUTO	1.721	*0.005*	1.827	*0.003*	23.673	0.063	1.848	0.210	2.204	*0.025*
ENST00000550337.1	2.023	*0.004*	1.925	*0.013*	2.984	0.132	33.73	0.184	4.026	*0.038*
CDKN2BAS1	3.173	*0.006*	4.925	*0.002*	4.188	0.068	2.741	0.100	2.995	*0.044*
lincRNA-p21	1.867	*0.021*	1.970	*0.033*	3.492	0.096	2.283	0.311	6.395	*0.013*
HOTAIR	4.348	*0.001*	5.256	*0.001*	2.556	0.342	1.651	0.524	8.125	*0.015*
GAS5	10.642	*0.001*	14.054	*0.001*	20.820	0.226	22.512	0.128	12.214	0.069
XIST	0.388	*0.003*	3.824	*0.004*	3.677	0.145	2.318	0.386	3.166	*0.024*
PANDA	7.960	*0.003*	15.737	*0.002*	30.052	*0.041*	27.726	0.151	3.548	0.253
NBR2	2.045	*0.041*	1.728	0.159	1.496	0.522	1.869	0.443	2.675	0.141
RNCR3	1.464	0.144	1.650	0.065	1.795	0.394	1.582	0.410	1.740	0.225
MIAT	6.591	*0.002*	5.293	*0.012*	8.383	0.235	8.753	0.181	5.388	0.118
MEG3	5.669	*0.013*	6.444	*0.017*	17.060	0.141	12.830	0.063	57.903	*0.023*
LET	7.116	*0.014*	5.806	*0.036*	6.736	0.068	4.399	0.534	2.079	0.584
MALAT1	9.945	*0.008*	5.156	*0.046*	4.712	0.193	12.858	0.343	33.033	0.086
GM4419	2.468	0.104	2.142	0.242	3.254	0.232	4.832	0.293	1.937	0.433
SALRNA1	0.161	*0.013*	0.114	*0.009*	0.029	0.127	0.072	0.057	0.092	0.100
THRIL	0.047	*0.001*	0.529	*0.001*	0.013	0.084	0.063	0.139	0.333	0.271

## Discussion

Recent literature implies that the dysregulation of lncRNA expression and functionality contributes to several pathophysiological states as several lncRNAs get validated as bona fide prognostic/diagnostic markers and drug targets [9, 18, 19]. The role of lncRNAs in the pathogenesis of type 2 diabetes mellitus and related complications has only recently been recognized, but there is already some evidence for their involvement in many of the pathophysiological mechanisms that are linked to the genesis and progression of disease [20, 21]. Despite the fact that Asian Indians are highly insulin resistant [10], more prone to develop type 2 diabetes mellitus (T2DM) and associated vascular complications [11], and exhibit increased susceptibility to early β-cell dysfunction [22], there is virtually lack of data on the role of lncRNAs in the clinical diabetes setting. Our study is the first report from India to show an association of altered signatures of lncRNAs in T2DM with pathological connectivity reflected by poor glycemic control, insulin resistance, accelerated cellular senescence, and meta-inflammation.

Our study is in consistent with the recent literature of several lncRNAs upregulated in diabetes state. In support of our findings, increased expression of GAS5 [23] and lncRNA ENST00000550337.1 [24] was reported in type 2 diabetes even with high diagnostic claim and biomarker value. A role for lncRNAs XIST [25] and GM4419 [26] was implicated in diabetic nephropathy while alterations in PANDA [27] and NBR2 [28] P21 [29] were linked to cellular senescence, AMPK regulation, and liver fibrosis, respectively. Expression levels of lncRNA-LET was shown to be decreased in a certain type of cancers [30], but we observed it to be upregulated in patients with type 2 diabetes. MIAT is identified to be involved in various diseases, particularly myocardial infarction, diabetic retinopathy, and various other microvascular complications [31]. Similarly, lncRNA RNCR3 was shown to be increased in retinal vasculature of an animal model as well as in vitro cell model [32]. While lncRNA PLUTO has been shown to be downregulated in islets from donors who are patients with type 2 diabetes and pre-diabetes subjects [33], our study observed a highly significant upregulation of PLUTO in patients with type 2 diabetes. Previous studies also reported that upregulated expression of lncRNA MALAT1 was linked to hyperglycemia-induced inflammation and endothelial dysfunction [34], diabetic nephropathy [35], and gestational diabetes mellitus [36]. In vitro studies demonstrated that HOTAIR interacts with the various chromatin-modifying enzymes and thereby participates in the regulation of gene expression [37]. A functional role for HOTAIR in the diabetes pathogenesis is yet to be established; however, its role has been hinted to be associated with regional adiposity [38]. lncRNA MEG3 has an important regulatory role in beta cell function [39], and the knock-down of MEG3 has been shown contributing to the pathology of diabetic microvascular complication [40]. In contrast, MEG3 gene expression was shown upregulated in the hepatocytes from mice fed with high-fat diet as well as in ob/ob mice and this has been linked to increased hepatic gluconeogenesis [41].

Our study provides the first preliminary evidence that expression of the long non-coding RNAs, THRIL, and SALRNA1 were decreased in patients with type 2 diabetes and negatively correlated with hyperglycemia, senescence, and inflammation. THRIL was shown to regulate TNF-α expression through an epigenetic mechanism, and TNF-α can also reduce THRIL expression via a negative feedback action [42]. Similarly, SAL-RNA1 was earlier identified as putative age-delaying lncRNA, since its reduction with small inhibitory RNAs (siRNA) induced rapid aging changes of the fibroblasts, such as large cell morphology, positive β-galactosidase activity, and upregulation of p53 [43]. Notably, lncRNA ANRIL shown upregulated in our study was also linked to CDKN2A/B, a strong type 2 diabetes risk gene variant [44, 45].

It is interesting to note that the majority of differentially expressed lncRNAs in patients with type 2 diabetes observed in our study are involved in cell cycle regulation and senescence and their expression levels correlated to poor glycemic control, insulin resistance, accelerated senescence, and inflammation. Several lncRNAs were reported to influence the molecular processes that underlie age-associated phenotypes and play an important role in accelerated aging [4, 46]. Type 2 diabetes has been linked to cellular senescence, senescence-associated secretory phenotype (SASP), and accelerated aging [47, 48], and our lab was the first one in the world literature to report an association of increased telomere shortening in patients with type 2 diabetes [2, 3]. Earlier, we have also shown increased HDAC3 epigenetic signature in patients with type 2 diabetes [49], and in the present study, there was a positive correlation of HDAC3 mRNA expression with majority of the lncRNAs and this endorses the concerted and coordinated interactions between lncRNAs and histone modifications [50].

Our work offers an avenue for several translational applications including a role of lncRNAs in lifestyle changes. Recent findings suggest a putative role of non-coding RNAs in physical activity and several miRNAs have been identified as modulators of exercise-induced adaption at both systemic and tissue levels [51]. Contrast to miRNAs, little is known about the role of long non-coding RNAs (lncRNAs) in exercise. Identification of the role of lncRNAs in exercise will improve our understanding of exercise physiology and has the potential to enhance the application of current therapeutic approaches. In fact, a micropeptide encoded by a putative lncRNA has been shown to regulate muscle performance [52]. Although very little is known about the relationship between lncRNAs and dietary factors, it appears that dietary manipulation could also beneficially alter the expression of lncRNAs and thereby ensure health [53].

One of the limitations of our study is of its cross-sectional nature as well as small sample size, and hence, the findings of the study and its conclusions should be interpreted with caution. From this pilot study, we could not extrapolate causal link of alterations in lncRNAs with type 2 diabetes, and it needs replication and prospective follow-up studies. Secondly, considering the tissue-specific and heterogeneous actions of lncRNAs, the alterations seen in PBMCs might only mirror disease-pathology directionality. However, the altered expression profile of lncRNAs in PBMCs has been shown to reflect the pathophysiology in different disease states including multiple sclerosis [54], myocardial infarction [55], and rheumatoid arthritis [56]. In fact, a recent study of deep RNA sequencing uncovered a repertoire of human macrophage lncRNAs modulated by macrophage activation and closely linked it to the pathophysiology of cardiometabolic diseases [57].

## Conclusion

To conclude, our study is of its first kind in India to report altered lncRNA profiles linked to poor glycemic control, insulin resistance, senescence, and proinflammation in patients with type 2 diabetes. A better understanding of the mechanisms underlying the functions of lncRNAs will help us to understand the ever-expanding pathophysiology of diabetes and its complications and thereby adapt to prevention strategies as well as to develop novel therapeutic agents.

## Additional files

Additional file 1: **Table S1.** Correlation analysis of LncRNAs with clinical and biochemical parameters. (DOCX 21 kb)

Additional file 2: **Table S2.** Correlation analysis of LncRNAs with molecular parameters. (DOCX 18 kb)

## Abbreviations

BMI: Body mass index
GLB1: β-Galactosidase 1
HbA1C: Glycated hemoglobin
HDAC: Histone deacetylase
HOMA-IR: Homeostatic model assessment-insulin resistance
IL-1β: Interleukin-1 beta
IL-6: Interleukin-6
lncRNAs: Long non-coding RNAs
MCP-1: Monocyte chemoattractant protein 1
NFκB: Nuclear factor kappa-light-chain-enhancer of activated B cells
NGT: Normal glucose tolerance
P16: Cyclin-dependent kinase inhibitor 2A
P21: Cyclin-dependent kinase inhibitor 1A
P53: Tumor protein/tumor suppressor 53
PBMCs: Peripheral blood mononuclear cells
SASP: Senescence-associated secretory phenotype
Sirt1: Sirtuin (silent mating-type information regulation 2 homolog) 1
SOCS-3: Suppressor of cytokine signaling 3
T2DM: Type 2 diabetes mellitus
TNF-α: Tumor necrosis factor-α
