# Supplementary material for: Linking a role of lncRNAs (long non-coding RNAs) with insulin resistance, accelerated senescence, and inflammation in patients with type 2 diabetes
Source: Hum Genomics. 2018 Aug 23;12:41. doi: 10.1186/s40246-018-0173-3 (PMC6107963; doi:10.1186/s40246-018-0173-3)
Supplement: Supplementary file 1 — Table S1. Correlation analysis of LncRNAs with clinical and biochemical parameters. (DOCX 21 kb) [file 40246_2018_173_MOESM1_ESM.docx]

**Additional file 1: Table S1. Correlation analysis of LncRNAs with clinical and biochemical parameters**

| Variables | PLUTO | ENST00000550337.1 | CDKN2BAS1 | LINCRNA-p21 | HOTAIR | GAS5 | XIST | PANDA | NBR2 | RNCR3 | MIAT | MEG3 | LET | MALAT1 | GM4419 | SALRNA1 | THRIL |
| --- | --- | --- | --- | --- | --- | --- | --- | --- | --- | --- | --- | --- | --- | --- | --- | --- | --- |
| Age (years) | *r=*-0.018 *p*=0.890 | 0.210 0.107 | 0.188 0.143 | 0.214 0.095 | 0.197 0.124 | -0.064 0.621 | 0.041 0.749 | -0.036 0.784 | 0.136 0.291 | 0.001 0.995 | 0.132 0.306 | 0.338 **0.007** | 0.109 0.397 | 0.188 0.144 | 0.038 0.772 | -0.251 **0.049** | 0.009 0.944 |
| BMI (kg/m^2^) | *r=*0.030 *p*=0.819 | 0.079 0.549 | 0.039 0.763 | 0.001 0.997 | 0.093 0.473 | 0.228 0.075 | 0.141 0.273 | 0.120 0.355 | 0.222 0.083 | -0.094 0.468 | 0.299 **0.018** | -0.117 0.366 | 0.246 **0.054** | 0.196 0.128 | 0.212 0.098 | 0.158 0.221 | -0.067 0.608 |
| Waist (cm) | *r=*-0.022 *p*=0.867 | 0.061 0.649 | 0.197 0.131 | -0.006 0.965 | 0.241 0.064 | 0.187 0.152 | 0.206 0.115 | 0.209 0.109 | 0.383 **0.002** | -0.109 0.409 | 0.410 **0.001** | 0.039 0.770 | 0.176 0.180 | 0.084 0.525 | 0.263 **0.042** | -0.030 0.817 | -0.128 0.329 |
| Sys BP(mm Hg) | *r=*-0.118 *p*=0.374 | 0.009 0.948 | 0.047 0.721 | -0.028 0.836 | -0.053 0.691 | 0.056 0.671 | 0.098 0.458 | 0.059 0.657 | 0.209 0.111 | -0.091 0.493 | 0.166 0.209 | 0.170 0.199 | 0.246 **0.061** | 0.205 0.118 | 0.185 0.160 | -0.162 0.219 | -0.130 0.327 |
| Dia BP (mm Hg) | *r=*-0.246 *p*=0.061 | -0.074 0.586 | 0.070 0.600 | -0.206 0.117 | -0.091 0.495 | -0.177 0.180 | -0.006 0.967 | 0.009 0.944 | 0.106 0.426 | -0.266 **0.042** | 0.100 0.449 | -0.057 0.670 | 0.260 **0.046** | 0.209 0.112 | 0.043 0.749 | -0.013 0.924 | -0.058 0.663 |
| FBS (mg/dl) | *r=*0.273 *p*=**0.032** | 0.344 **0.007** | 0.336 **0.008** | 0.279 **0.028** | 0.291 **0.022** | 0.349 **0.005** | 0.336 **0.008** | 0.344 **0.006** | 0.273 **0.032** | 0.131 0.308 | 0.389 **0.002** | 0.330 **0.009** | 0.367 **0.003** | 0.331 **0.009** | 0.223 0.081 | -0.216 0.092 | -0.305 **0.016** |
| HbA1c (%) | *r=*0.285 *p*=**0.025** | 0.434 **0.001** | 0.410 **0.001** | 0.479 **0.001** | 0.337 **0.007** | 0.338 **0.007** | 0.301 **0.017** | 0.265 **0.038** | 0.151 0.241 | 0.130 0.312 | 0.315 **0.012** | 0.266 **0.037** | 0.290 **0.022** | 0.291 **0.022** | 0.066 0.609 | -0.278 **0.029** | -0.259 **0.042** |
| HOMA-IR | *r=*0.237 *p*=0.063 | 0.346 **0.007** | 0.407 **0.001** | 0.166 0.198 | 0.362 **0.004** | 0.349 **0.005** | 0.394 **0.002** | 0.443 **0.001** | 0.212 0.098 | 0.149 0.249 | 0.354 **0.005** | 0.420 **0.001** | 0.326 **0.010** | 0.287 **0.023** | 0.143 0.269 | -0.210 0.101 | -0.262 **0.040** |
| Fasting insulin (µIU/ml) | *r=*0.226 *p*=0.078 | 0.330 **0.010** | 0.415 **0.001** | 0.113 0.383 | 0.334 **0.008** | 0.450 **0.001** | 0.367 **0.003** | 0.456 **0.001** | 0.172 0.182 | 0.132 0.308 | 0.342 **0.007** | 0.332 **0.008** | 0.349 **0.005** | 0.280 **0.027** | 0.170 0.188 | -0.258 **0.043** | -0.267 **0.036** |
| CHO (mg/dl) | *r=*0.137 *p*=0.287 | 0.033 0.803 | 0.025 0.850 | 0.189 0.142 | 0.044 0.737 | -0.112 0.384 | 0.049 0.708 | -0.268 **0.035** | -0.048 0.713 | 0.233 0.069 | -0.191 0.137 | -0.080 0.538 | -0.065 0.618 | 0.014 0.915 | -0.076 0.557 | 0.009 0.947 | 0.156 0.226 |
| TG (mg/dl) | *r=*-0.121 *p*=0.349 | -0.033 0.802 | -0.040 0.760 | -0.142 0.270 | -0.126 0.737 | -0.276 **0.030** | 0.081 0.532 | -0.106 0.411 | 0.273 **0.032** | -0.169 0.189 | 0.177 0.168 | -0.022 0.866 | 0.058 0.652 | 0.126 0.328 | 0.209 0.103 | 0.204 0.112 | -0.078 0.548 |
| HDL (mg/dl) | *r=*0.058 *p*=0.657 | 0.130 0.322 | -0.039 0.763 | 0.242 0.058 | 0.102 0.432 | 0.025 0.850 | -0.145 0.261 | -0.241 0.059 | -0.181 0.159 | 0.086 0.504 | -0.239 0.061 | -0.162 0.210 | -0.199 0.121 | -0.304 **0.016** | -0.216 0.092 | -0.268 **0.035** | 0.140 0.279 |
| LDL (mg/dl) | *r=*0.194 *p*=0.130 | 0.012 0.925 | 0.057 0.662 | 0.207 0.106 | 0.075 0.563 | -0.018 0.889 | 0.064 0.619 | -0.193 0.133 | -0.118 0.359 | 0.318 **0.012** | -0.227 0.076 | -0.034 0.791 | -0.038 0.767 | 0.053 0.684 | -0.114 0.378 | 0.003 0.981 | 0.174 0.177 |
| VLDL | *r=*-0.121 *p*=0.357 | -0.037 0.780 | -0.038 0.772 | -0.142 0.279 | -0.130 0.321 | -0.278 **0.031** | 0.083 0.526 | -0.105 0.425 | 0.274 **0.034** | -0.175 0.182 | 0.188 0.151 | -0.022 0.868 | 0.054 0.683 | 0.128 0.330 | 0.209 0.109 | 0.202 0.121 | -0.082 0.534 |
