# Supplementary material for: Linking a role of lncRNAs (long non-coding RNAs) with insulin resistance, accelerated senescence, and inflammation in patients with type 2 diabetes
Source: Hum Genomics. 2018 Aug 23;12:41. doi: 10.1186/s40246-018-0173-3 (PMC6107963; doi:10.1186/s40246-018-0173-3)
Supplement: Supplementary file 2 — Table S2. Correlation analysis of LncRNAs with molecular parameters. (DOCX 18 kb) [file 40246_2018_173_MOESM2_ESM.docx]

**Supplement Table 1b: Correlation analysis of LncRNAs with molecular parameters**

| Variables | PLUTO | ENST00000550337.1 | CDKN2BAS1 | LINCRNA-p21 | HOTAIR | GAS5 | XIST | PANDA | NBR2 | RNCR3 | MIAT | MEG3 | LET | MALAT1 | GM4419 | SALRNA1 | THRIL |
| --- | --- | --- | --- | --- | --- | --- | --- | --- | --- | --- | --- | --- | --- | --- | --- | --- | --- |
| HDAC3 | *r*=0.250 *p*=**0.050** | 0.092 0.483 | 0.282 **0.027** | 0.212 0.098 | 0.300 **0.018** | 0.450 **0.001** | 0.397 **0.001** | 0.340 **0.007** | 0.215 0.093 | 0.143 0.269 | 0.367 **0.003** | 0.205 0.110 | 0.286 **0.065** | 0.263 **0.039** | 0.236 0.064 | -0.159 0.216 | -0.283 **0.026** |
| Sirt1 | *r*=-0.029 *p*=0.825 | -0.179 0.170 | -0.174 0.175 | -0.045 0.729 | -0.255 **0.045** | -0.084 0.517 | -0.216 0.091 | -0.215 **0.093** | -0.083 0.521 | 0.095 0.463 | -0.184 0.152 | -0.293 0.061 | -0.162 0.208 | -0.189 0.141 | -0.127 0.325 | 0.060 0.643 | 0.143 0.266 |
| GLB1 | *r*=-0.102 *p*=0.431 | 0.013 0.923 | 0.158 0.221 | -0.036 0.779 | 0.360 **0.004** | 0.222 0.083 | 0.121 0.349 | 0.302 **0.017** | 0.367 **0.003** | -0.104 0.422 | 0.494 **0.001** | 0.072 0.577 | 0.393 **0.002** | 0.222 **0.083** | 0.372 **0.003** | 0.027 0.832 | -0.209 0.103 |
| P53 | *r*=0.023 *p*=0.860 | 0.089 0.500 | 0.302 **0.017** | 0.063 0.627 | 0.440 **0.001** | 0.273 **0.032** | 0.185 0.150 | 0.267 **0.036** | 0.203 0.113 | -0.018 0.890 | 0.421 **0.001** | 0.205 0.109 | 0.457 **0.001** | 0.270 **0.034** | 0.240 0.060 | -0.041 0.750 | -0.255 **0.045** |
| P21 | *r*=0.134 *p*=0.299 | 0.167 0.202 | 0.050 0.702 | 0.057 0.662 | 0.227 0.076 | 0.227 0.076 | 0.181 0.160 | 0.400 **0.001** | 0.053 0.680 | -0.031 0.811 | 0.122 0.344 | 0.337 **0.007** | 0.083 0.523 | -0.045 0.729 | -0.044 0.734 | -0.292 **0.021** | -0.394 **0.002** |
| P16 | *r*=0.095 *p*=0.462 | 0.203 0.120 | 0.473 **0.001** | 0.328 **0.009** | 0.278 **0.029** | 0.266 **0.037** | 0.259 **0.042** | 0.265 **0.038** | 0.094 0.470 | 0.075 0.561 | 0.241 0.059 | 0110 0.396 | 0.269 **0.035** | 0.255 **0.045** | 0.014 0.912 | -0.158 0.221 | -0.085 0.513 |
| TNF-α | *r*=0.266 *p*=**0.036** | 0.339 **0.008** | 0.346 **0.006** | 0.131 0.311 | 0.293 **0.021** | 0.323 **0.010** | 0.234 0.067 | 0.408 **0.001** | 0.169 0.188 | 0.121 0.349 | 0.373 **0.003** | 0.130 0.315 | 0.286 **0.024** | 0.337 **0.007** | 0.101 0.436 | -0.291 **0.022** | -0.461 **0.001** |
| IL6 | *r*=0.020 *p*=0.876 | 0.177 0.374 | 0.293 **0.021** | 0.103 0.424 | 0.269 **0.035** | 0.320 **0.011** | 0.171 0.184 | 0.248 **0.052** | 0.010 0.941 | -0.072 0.579 | 0.049 0.707 | 0.190 0.139 | 0.184 0.151 | 0.035 0.786 | -0.130 0.314 | -0.243 0.057 | -0.172 0.182 |
| MCP1/CCL2 | *r*=0.108 *p*=0.405 | 0.088 0.503 | 0.049 0.708 | 0.098 0.449 | -0.015 0.906 | 0.249 **0.051** | 0.093 0.474 | 0.411 **0.001** | -0.185 0.150 | 0.059 0.647 | -0.056 0.665 | 0.510 **0.001** | 0.239 **0.061** | 0.105 0.415 | -0.065 0.614 | -0.239 0.061 | -0.201 0.118 |
| IL1-β | *r*=0.154 *p*=0.233 | 0.137 0.298 | 0.299 **0.018** | 0.220 0.085 | 0.239 0.061 | 0.298 **0.019** | 0.164 0.202 | 0.296 **0.019** | 0.068 0.599 | 0.086 0.508 | -0.011 0.930 | 0.214 0.095 | 0.148 0.251 | 0.034 0.794 | -0.071 0.583 | -0.167 0.194 | -0.336 **0.008** |
| SOCS3 | *r*=0.238 *p*=0.063 | 0.079 0.548 | 0.110 0.394 | 0.212 0.098 | 0.366 **0.003** | 0.286 **0.024** | 0.177 0.169 | 0.332 **0.008** | 0.191 0.138 | 0.148 0.252 | 0.135 0.297 | 0.193 0.132 | 0.056 0.663 | 0.096 0.459 | 0.161 0.212 | -0.067 0.605 | -0.381 **0.002** |
| Telomere length | *r*=-0.240 *p*=0.060 | -0.339 **0.008** | -0.215 0.093 | -0.223 0.081 | -0.352 **0.005** | -0.299 **0.018** | -0.284 **0.025** | -0.312 **0.013** | -0.297 **0.019** | -0.080 0.537 | -0.362 **0.004** | -0.232 0.069 | -0.363 **0.004** | -0.333 **0.008** | -0.198 0.123 | 0.141 0.273 | 0.388 **0.002** |
